# Supplementary material for: Genetic and immune determinants of E. coli liver abscess formation
Source: Proc Natl Acad Sci U S A. 2023 Dec 14;120(51):e2310053120. doi: 10.1073/pnas.2310053120 (PMC10743367; doi:10.1073/pnas.2310053120)
Supplement: Supplementary file 1 — Appendix 01 (PDF) [file pnas.2310053120.sapp.pdf]

Supporting Information for
Genetic and immune determinants of *E. coli* liver abscess formation

Karthik Hullahalli, Katherine G. Dailey, Yuko Hasegawa, Encarnacion Torres, Masataka Suzuki, Hailong Zhang, David W. Threadgill, Victor M. Navarro, and Matthew K. Waldor

Address correspondence to KH (hullahalli@g.harvard.edu) or MKW (mwaldor@bwh.harvard.edu)

This PDF file includes:

Figures S1 to S12
Legend for Dataset S1

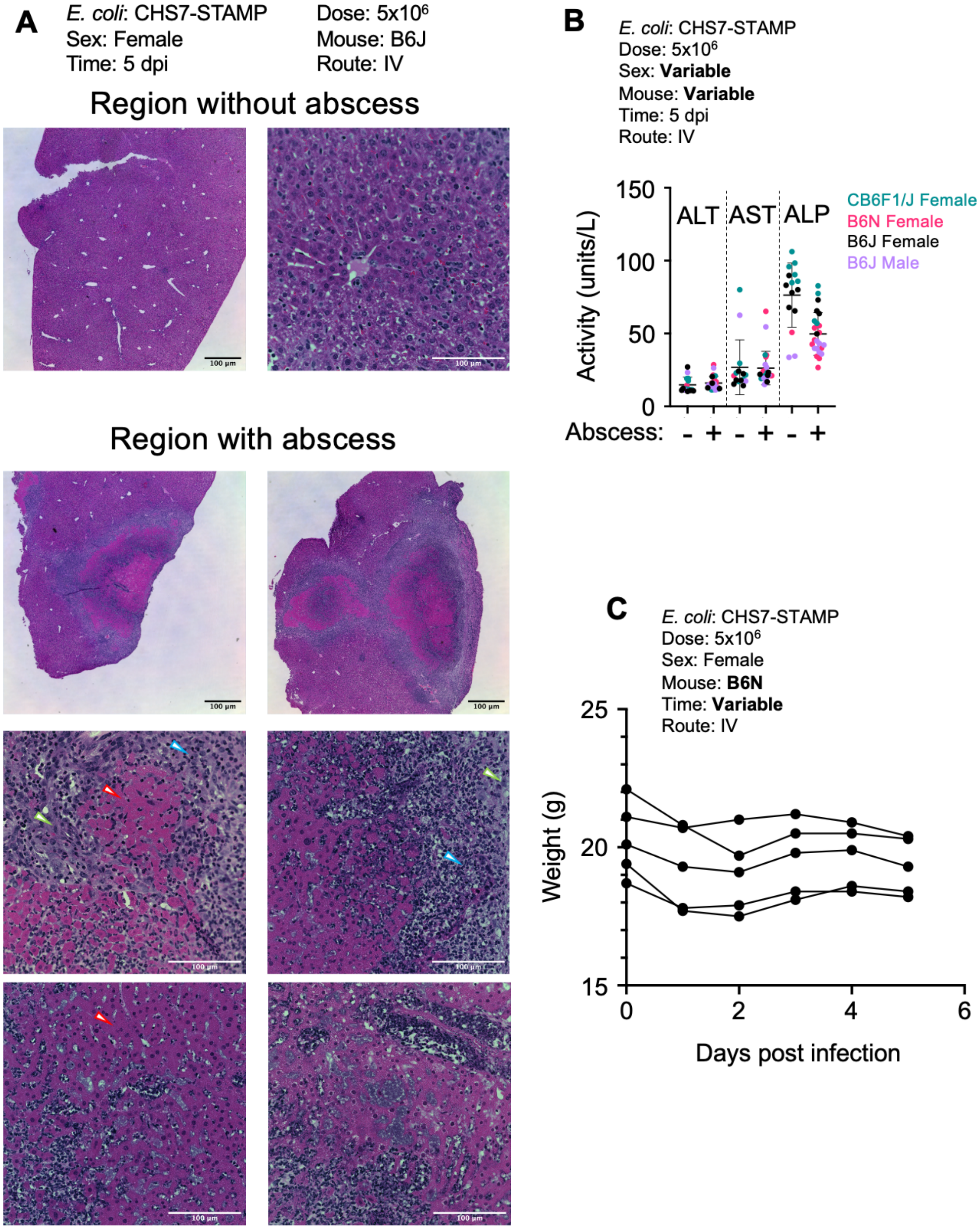

Figure S1. Histology and health outcomes of *E. coli*-induced liver abscesses A) H&E staining of abscesses in B6J females. Necrotic hepatocytes (deep pink stain, red arrowheads) are surrounded by cells that resemble macrophages (green arrowhead) and neutrophils (blue arrowhead). B) Serum levels of alanine aminotransferase (ALT), aspartate aminotransferase (AST), and alkaline phosphatase (ALP) are similar in mice that possess or lack abscesses. Serum was collected from the same mice used in Figure 2 and Figure 4. C) B6N female mice exhibit early weight loss up to 2 dpi and then stabilize in weight.

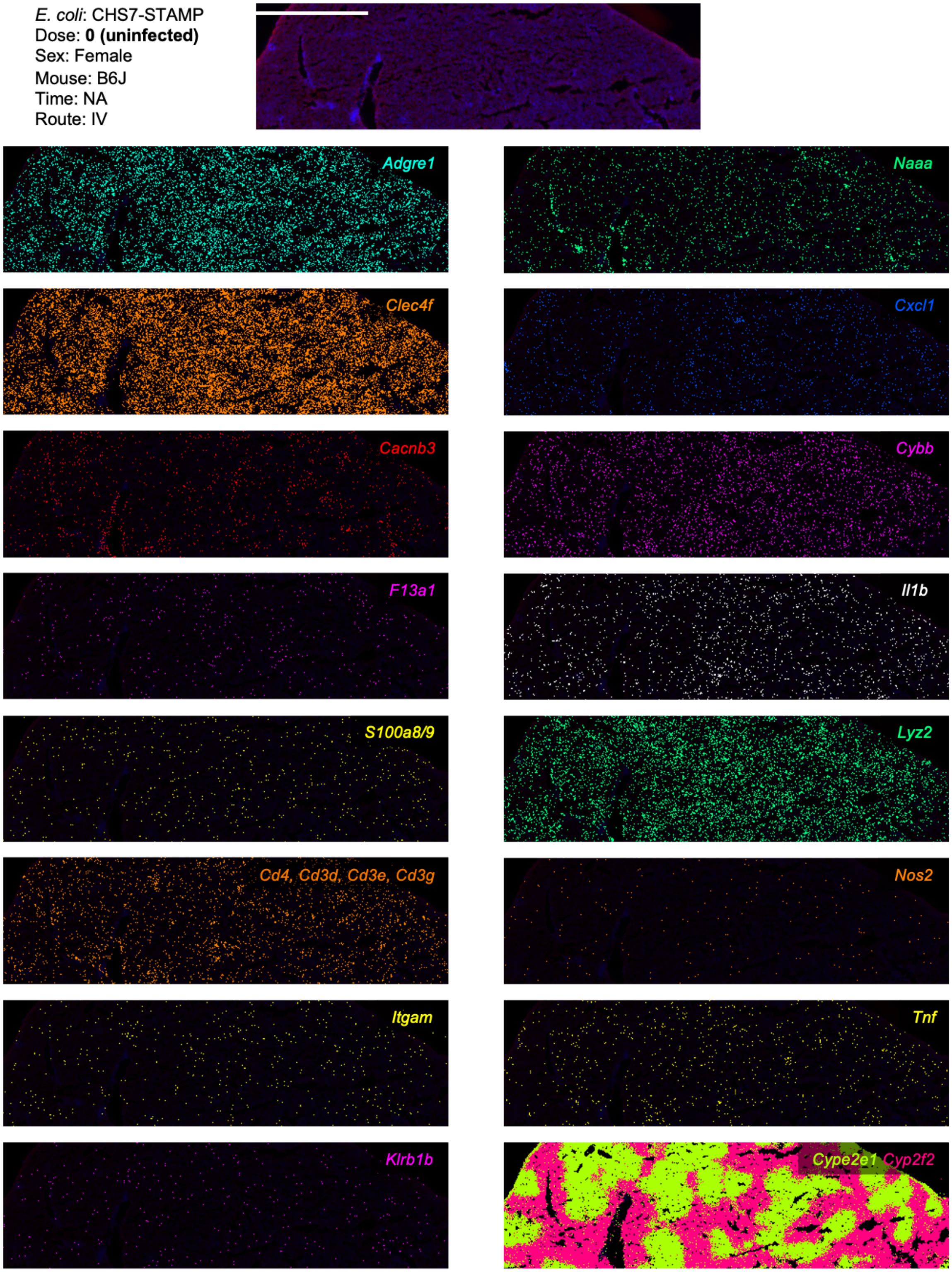

Figure S2. Spatial transcriptomic profile of uninfected liver tissue. MERSCOPE images of liver from uninfected samples from Figure 3 but individual genes are shown here separately. Cell types and corresponding transcripts are macrophages (*Adgre1),* migratory dendritic cells (*Cacnb3*), Kupffer cells (*Clec4f*), monocytes (*F13a1*), neutrophils (*S100a8/9*), T cells (*Cd4, Cd3g, Cd3e,* and *Cd3b*), various leukocytes (*Itgam*, also known as CD11b), NK/ILC1s (*Klrb1b*), and cDC1s (*Naaa*).

Cxcl1, Cybb, Il1b, Lyz2, Nos2, and *Tnf* correspond to known markers of inflammatory responses. *Cyp2e1* and *Cyp2f2* correspond to markers of hepatocyte zonation.

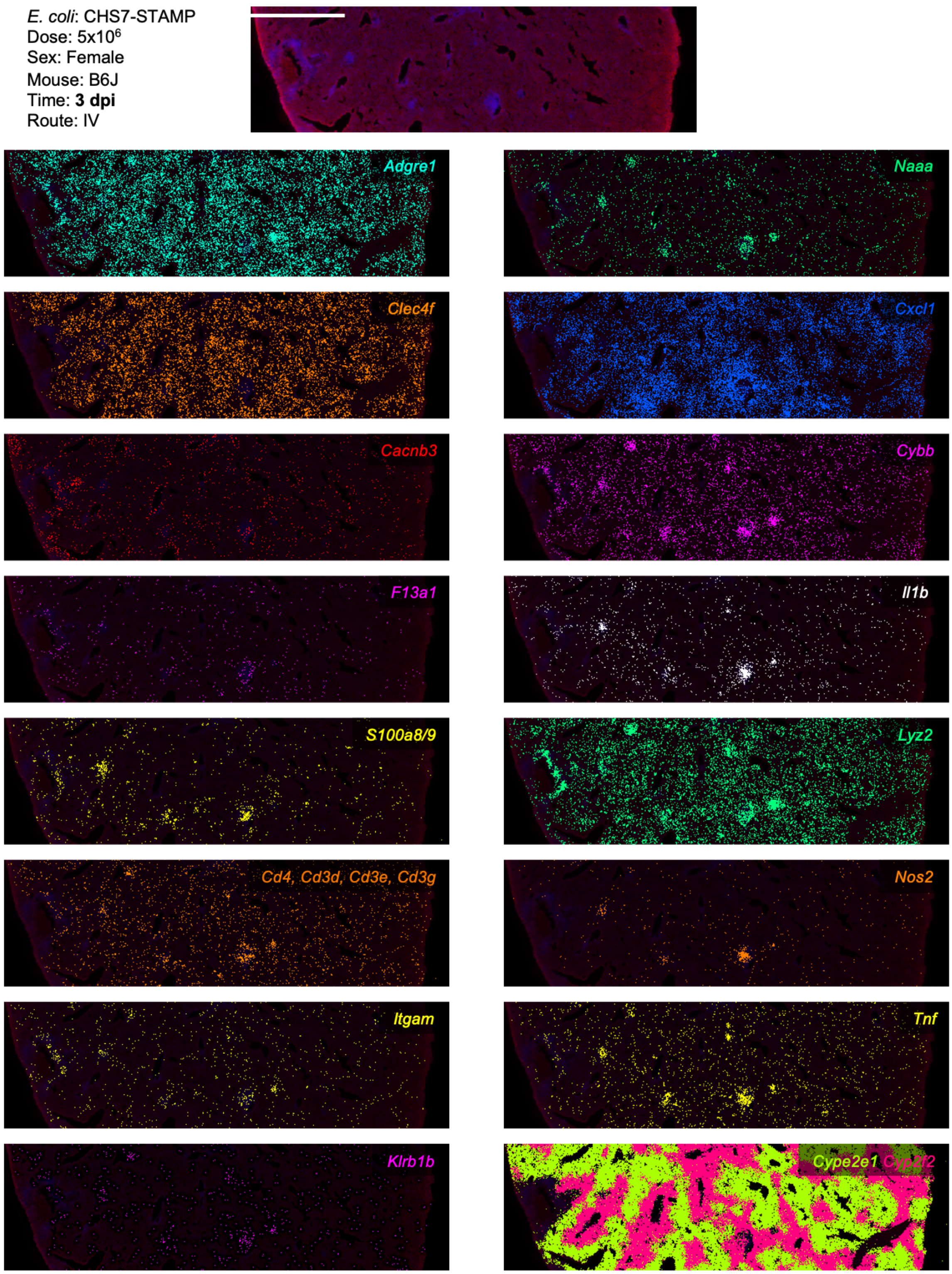

Figure S3. Spatial transcriptomic profile of 3 dpi abscess. MERSCOPE images of liver from 3 dpi samples from Figure 3 but individual genes are shown here separately. Cell types and corresponding transcripts are macrophages (*Adgre1),* migratory dendritic cells (*Cacnb3*), Kupffer cells (*Clec4f*), monocytes (*F13a1*), neutrophils (*S100a8/9*), T cells (*Cd4, Cd3g, Cd3e,* and *Cd3b*), various leukocytes (*Itgam*, also known as CD11b), NK/ILC1s (*Klrb1b*), and cDC1s (*Naaa*). *Cxcl1,*

Cybb, Il1b, Lyz2, Nos2, and *Tnf* correspond to known markers of inflammatory responses. *Cyp2e1* and *Cyp2f2* correspond to markers of hepatocyte zonation.

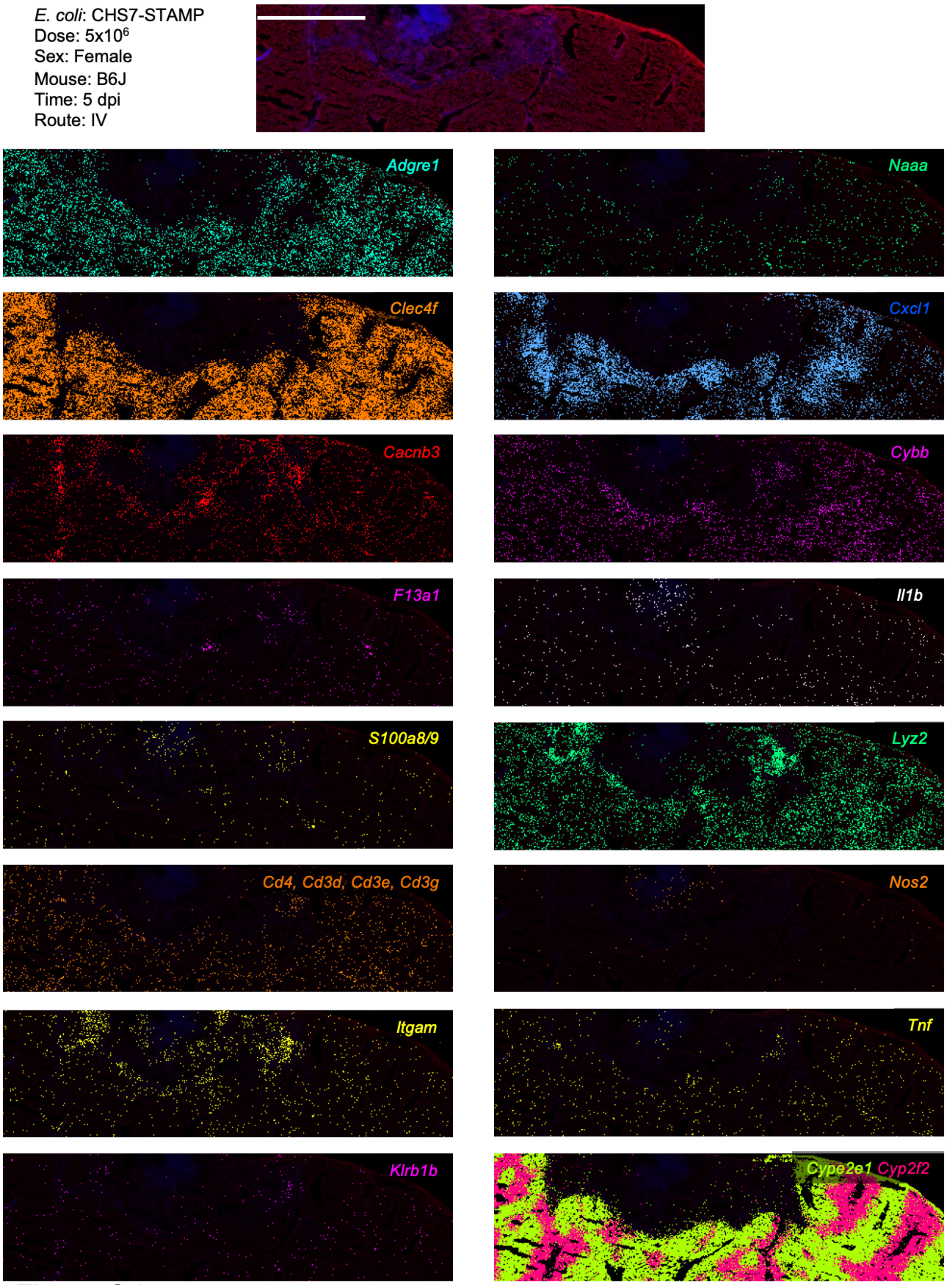

Figure S4. Spatial transcriptomic profile of 5 dpi abscess. MERSCOPE images of liver from 5 dpi samples from Figure 3 but individual genes are shown here separately. Cell types and corresponding transcripts are macrophages (*Adgre1),* migratory dendritic cells (*Cacnb3*), Kupffer cells (*Clec4f*), monocytes (*F13a1*), neutrophils (*S100a8/9*), T cells (*Cd4, Cd3g, Cd3e,* and *Cd3b*),

various leukocytes (*Itgam*, also known as CD11b), NK/ILC1s (*Klrb1b*), and cDC1s (*Naaa*). *Cxcl1, Cybb, Il1b, Lyz2, Nos2,* and *Tnf* correspond to known markers of inflammatory responses. *Cyp2e1* and *Cyp2f2* correspond to markers of hepatocyte zonation.

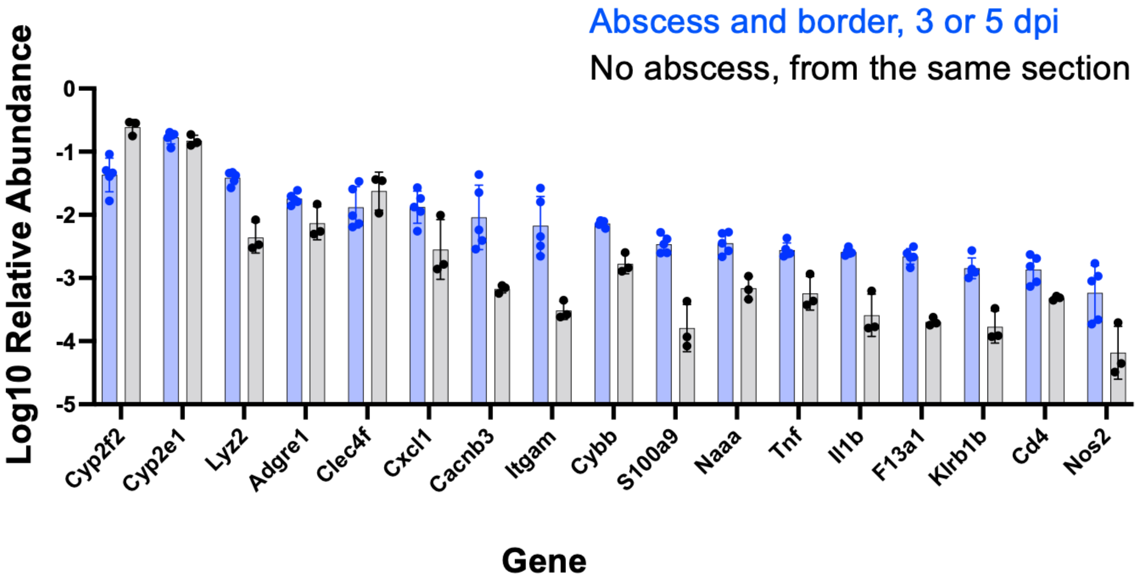

Figure S5. Quantification of MERSCOPE data. Quantification of relative transcript abundances within abscesses. Regions of interest were drawn around an abscess and bordering regions (blue) or control regions from the same section that lacked immune cell clusters (black). Transcripts were quantified relative to the total number of transcripts in the region. Data are derived from 3 and 5 dpi samples from 3 sections across two animals.

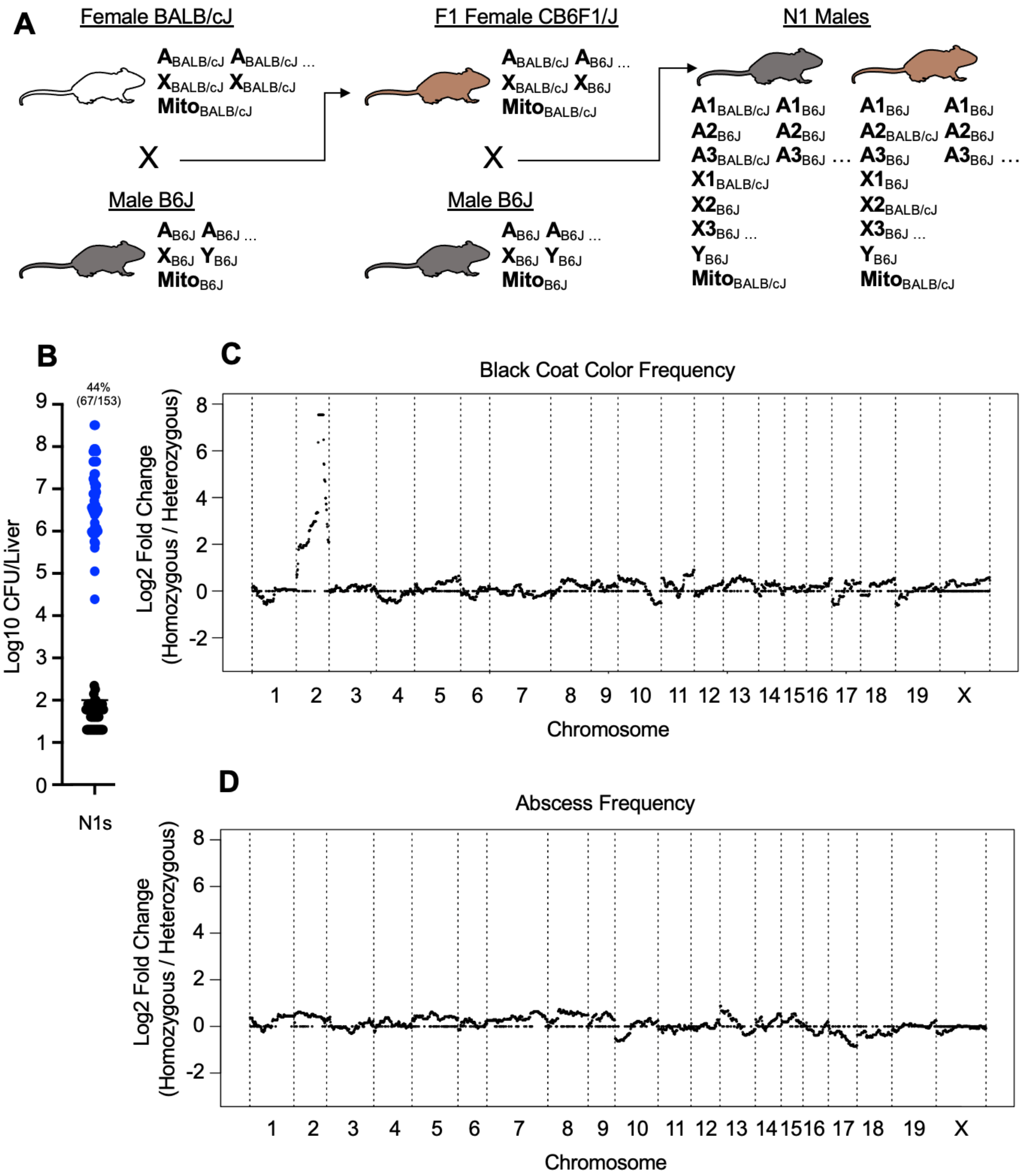

Figure S6. Backcross analysis of inheritance of abscess susceptibility. A) CB6F1/J heterozygotes were bred to male B6J mice to generate N1 backcross mice. The abscess-resistant phenotype of male heterozygous mice was expected to revert to susceptible after backcrossing with susceptible B6J mice. Therefore, only males that are homozygous B6J for the causal allele should develop abscesses. B) 44% of male N1 mice developed abscesses (blue). C) The agouti locus is identified when calculating the frequency of mice with black coat colors in homozygotes, relative to the frequency of mice with black coat color in heterozygotes. D) Same as C) but for abscess frequency instead of coat color. No association was observed between abscess susceptibility and B6J homozygosity.

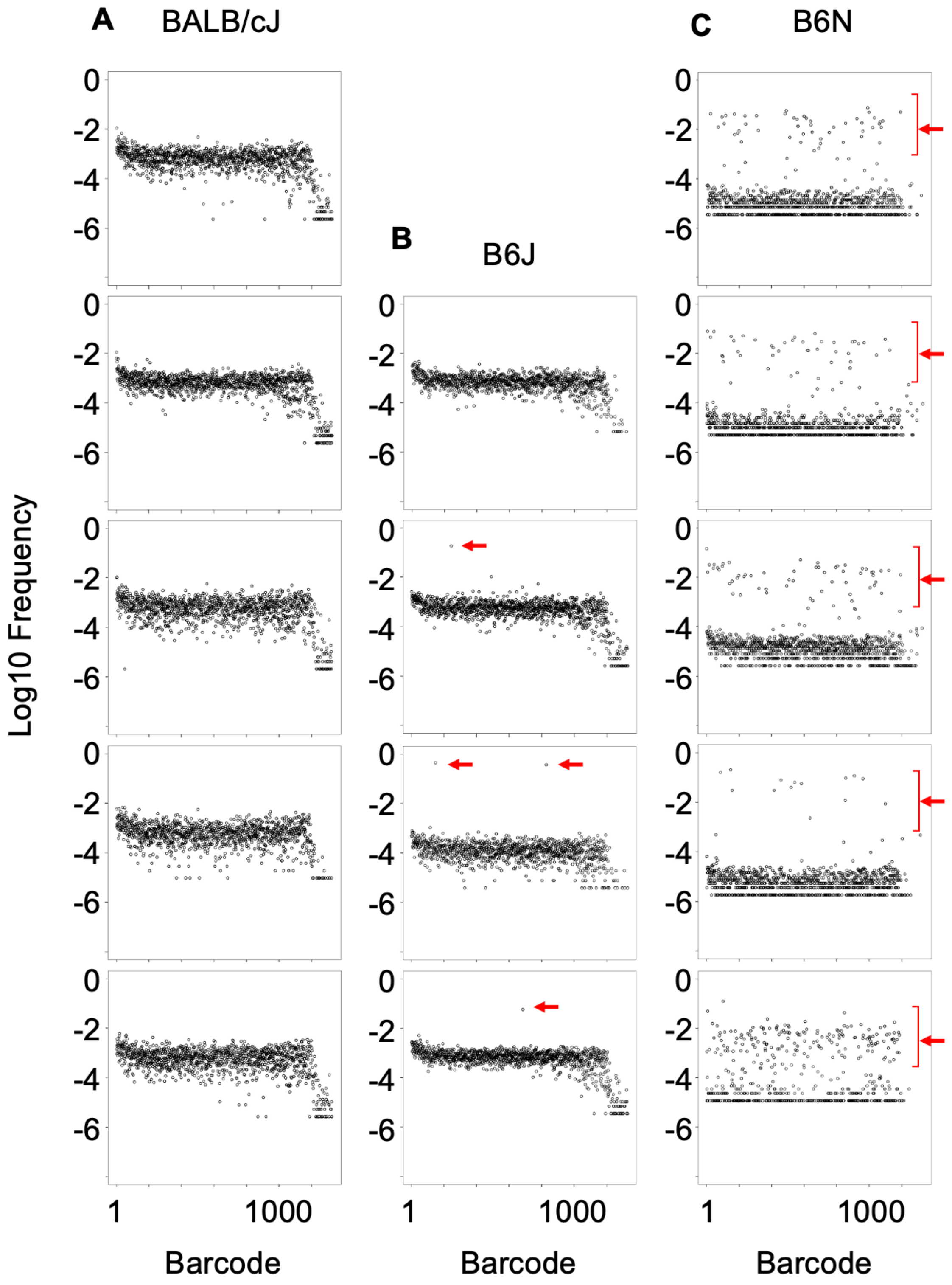

Figure S7. Clonal replication at 1 dpi correlates with abscess frequency. Data are replicate animals from Figure 5C. The X-axis is an arbitrary designation for barcode identity, and the Y-axis represents the relative frequency of each barcode. Red arrows denote replicated clones. A, B, and C correspond to BALB/cJ, B6J and B6N mice, respectively.

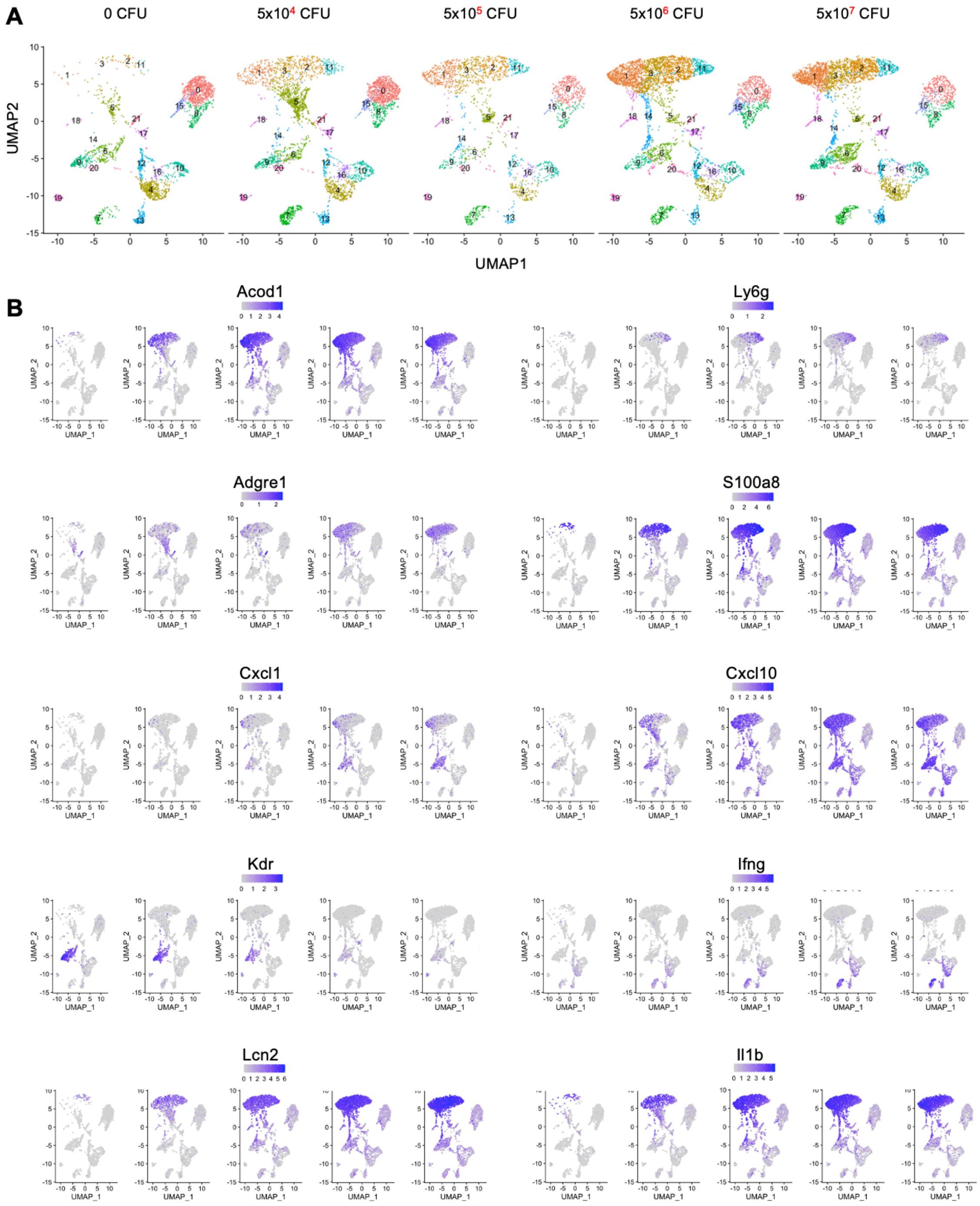

Figure S8. Additional genes from single cell RNA-sequencing of liver immune cells at 4hpi. A) UMAP plots are shown from Figure 6 for reference. B) Individual genes are shown as indicated from normalized expression data (sctransform).

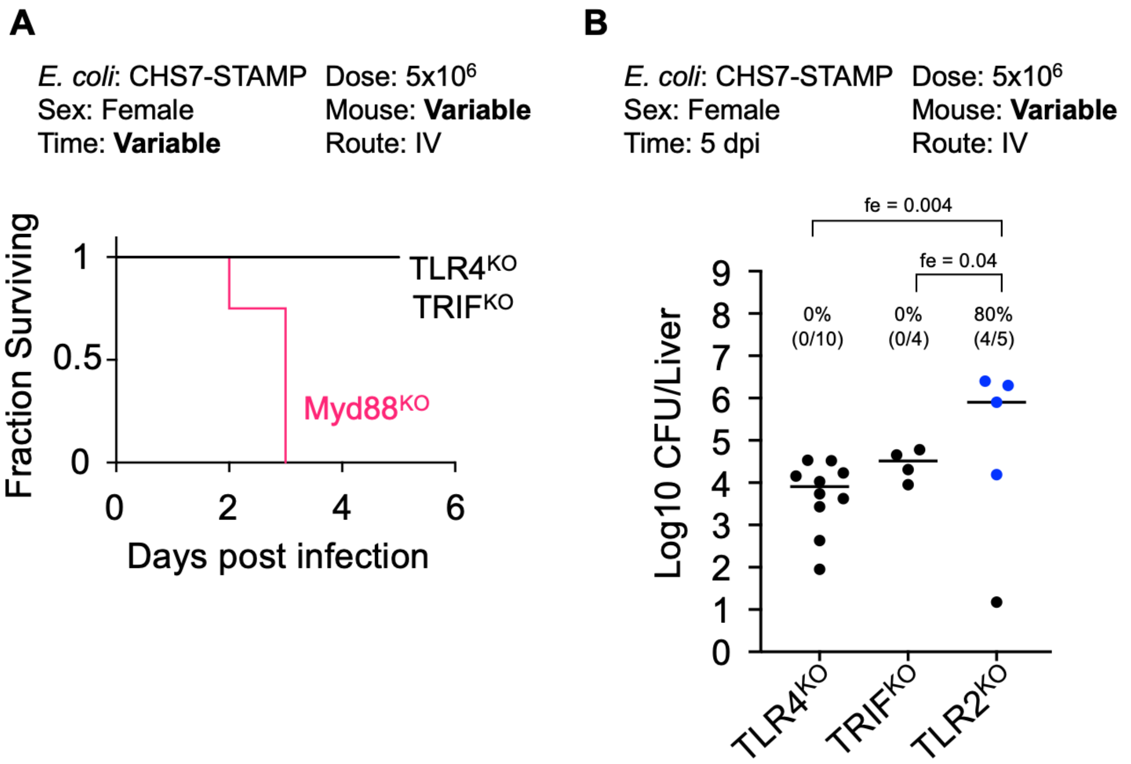

Figure S9. Mice lacking TRIF, but not mice lacking TLR2 and Myd88, phenocopy TLR4^{KO} mice. A) Myd88^{KO} (n=4) mice succumb to infection without abscess development, but TLR4^{KO} (n=10) and TRIF^{KO} (n=4) mice survive until 5 dpi. B) TRIF^{KO} mice phenocopy TLR4^{KO} mice both in the lack of abscess formation and the elevated CFU. In contrast, TLR2^{KO} mice are sensitive to abscess formation. P values are derived from one tailed Fisher Exact tests (fe).

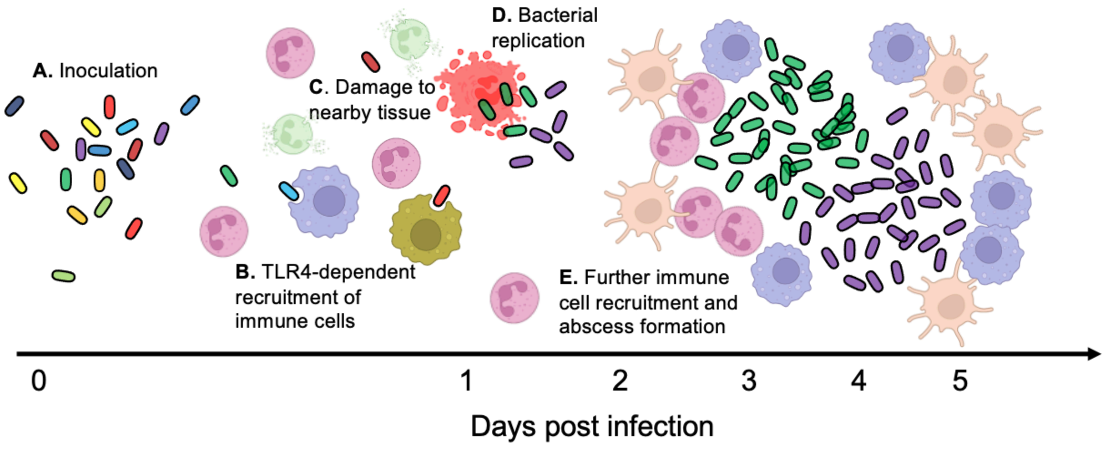

Figure S10. Proposed model for *E. coli* liver abscess formation. A) Inoculation of bacteria leads to the rapid recruitment of immune cells to the liver in a TLR4-dependent manner (B). Recruited inflammatory cells cause damage to neighboring tissue (C). *E. coli* exploits the newly necrotic niche to replicate by one day post inoculation (D), which leads to further recruitment of inflammatory cells and pathogen replication, until the abscess is fully formed (E).

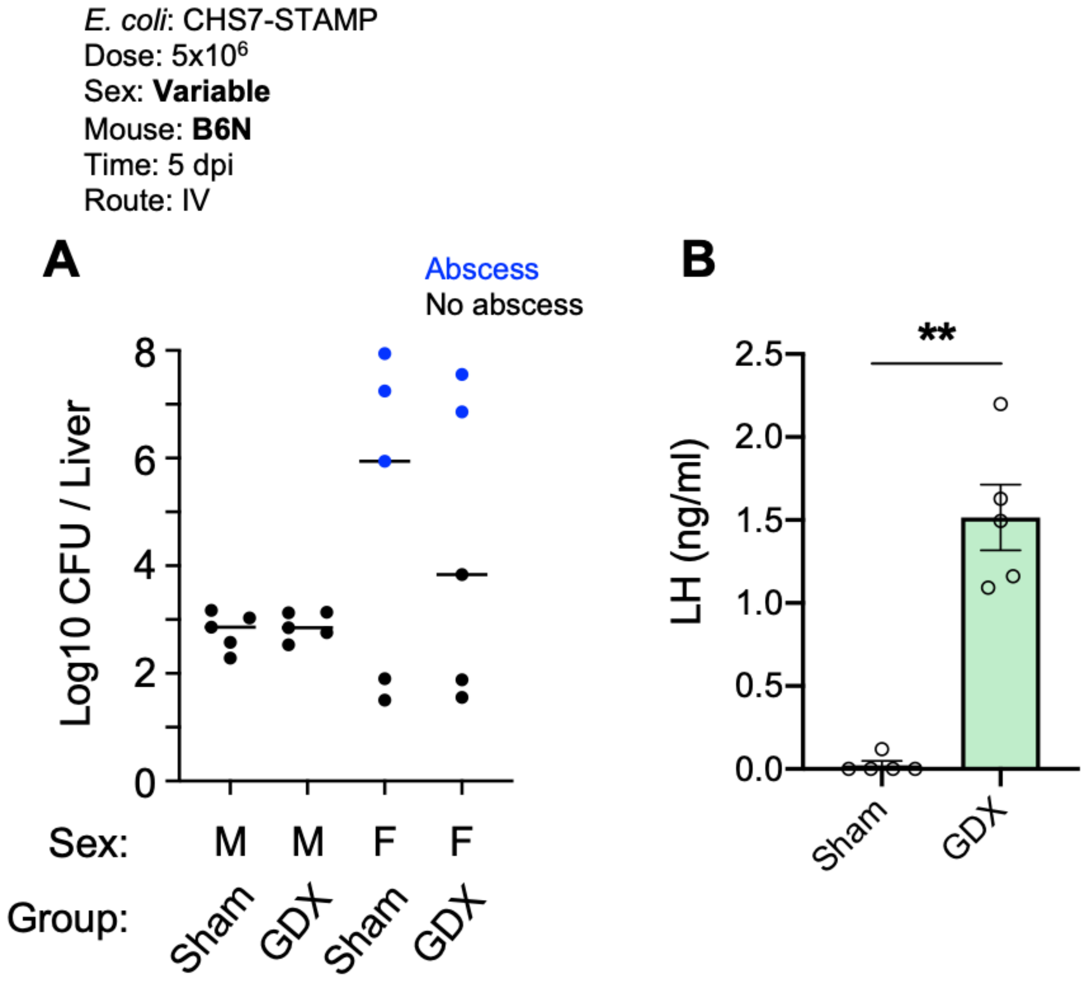

Figure S11. Gonadectomy at 10 days prior to infection does not alter abscess susceptibility. A) B6N males and females were subject to gonadectomy or sham surgery 10 days prior to infection. No change in abscess susceptibility was observed. B) Measurement of luteinizing hormone (LH) concentration by ELISA in males at 5 dpi confirmed successful depletion of sex steroids after gonadal removal. Mean and standard deviation are displayed. **p < 0.01 from one tailed students T-test.

E. coli: **Variable**
Dose: **Variable**
Sex: **Variable**
Mouse: **Variable**
Time: 5 dpi
Route: IV

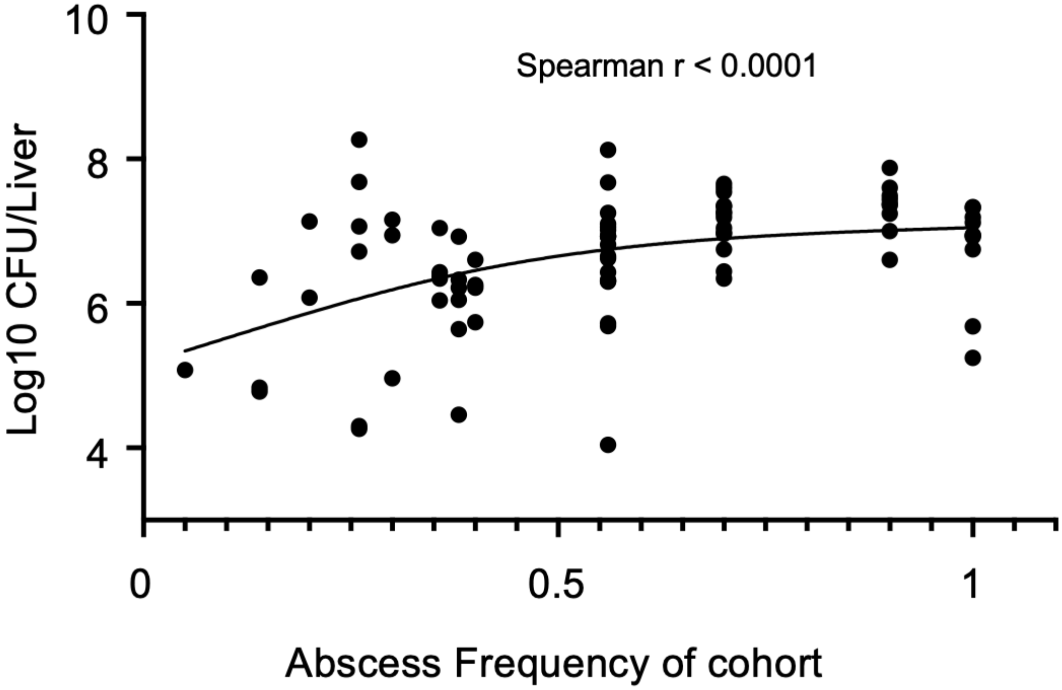

Figure S12. Abscess frequency is correlated with CFU. From every experiment in this study that assessed abscess frequency at 5 dpi, we plotted the CFU of each animal that developed abscesses as a function of the frequency of the abscess within the experimental cohort. Each point represents a single animal that developed abscesses, and animals within these cohorts that did not develop abscesses are not plotted. A 3-knotted smoothing spline is shown for visualizing the correlation. Both variables are positively correlated (Spearman r < 0.0001)

Dataset S1 (separate file). Genes in MERSCOPE panel
